# *Meloidogyne floridensis* has a unique virulence profile against root-knot nematode resistant and susceptible pepper (*Capsicum annuum*) lines

**DOI:** 10.2478/jofnem-2025-0007

**Published:** 2025-03-14

**Authors:** C Khanal, W Rutter, M. S Alam, I. Alarcon-Mendoza

**Affiliations:** Clemson University, Department of Plant and Environmental Sciences, 105 Collings St., Clemson, SC 29634; USDA-ARS, U.S. Vegetable Laboratory, 2700 Savannah Hwy., Charleston, SC 29449; University of California Davis, Department of Entomology and Nematology, Davis, CA 95616

**Keywords:** *Capsicum annuum*, host resistance, *Meloidogyne floridensis*, root-knot nematode, pepper

## Abstract

*Meloidogyne floridensis* is a recently described species of root-knot nematode (RKN) that is very closely related to many other tropical RKN species, including *M. incognita.* Despite its close phylogenetic relationship, *M. floridensis* is distinctive from its close relatives in both its meiotic mechanism of reproduction and its documented virulence on many of the most common RKN resistance genes in cultivated peach, tomato, and pepper. To further characterize the virulence profile of *M. floridensis*, we conducted replicate screens using this nematode to infect a panel of pepper lines that carry different sets of known RKN resistance genes. We found that *M. floridensis* was virulent against all the most common RKN resistance genes, including *N*, *Me1*, and *Me3*. We also found that two of these lines, PA 136 and PM 217, were highly resistant to *M. floridensis*. PA136 was previously considered to be universally susceptible to all other RKN species. Further testing of an F1 hybrid of this line confirmed this result and indicated that PA 136 contains a yet uncharacterized and potentially dominant source of species-specific resistance against *M. floridensis*. These surprising results provide additional data on the differences between *M. floridensis* and its close relatives, and identify new sources of resistance that could be used by pepper breeding programs to develop new cultivars with resistance against this nematode.

The peach root-knot nematode (*Meloidogyne floridensis*) is a unique and virulent species of root-knot nematode (RKN) that was first described in the state of Florida just 20 years ago (Handoo et al., 2004). Since then, *M. floridensis* has been reported in other states, including Georgia (Marquez et al., 2021), South Caroliana (Reighard et al., 2019), and California (Westphal et al., 2019). This species first gained attention for its ability to infect and damage RKN resistant peach rootstocks carrying the *RMia* resistance gene (Handoo et al., 2004, Maquilan 2018). *Meloidogyne floridensis* has now been found infecting other RKN resistant crop varieties, including almond carrying the *RMja* gene (Duval et al., 2019), tomato carrying the *Mi1.2* gene (Marquez & Hajihassani, 2023), and pepper carrying the N resistance gene (Stanley et al., 2009). Despite its ability to overcome several different RKN resistance genes, *M. floridensis* is very closely related to many of the most damaging and widespread tropical RKN species, including *M. incognita*, *M. javanica*, and *M. arenaria* (Powers et al., 2018). Unlike its close relatives however, *M. floridensis* is currently the only known diploid and meiotically parthenogenic species within the RKN Clade I (Szitenberg et al., 2017, Castagnone-Sereno et al., 2013). The virulence of *M. floridensis*, its close relationship to other damaging RKN species, and its unique lifestyle, makes it a species of concern for growers in the Southern US.

Pepper (*Capsicum* spp.) is a popular solanaceous crop in regions of the Southern US where *M. floridensis* is known to be present. Pepper is susceptible to infection and damage by many RKN species, and several different RKN resistance genes have been identified and incorporated into cultivated pepper lines. The three most common pepper resistance genes *N*, *Me1*, and *Me3* all confer resistance to the same cosmopolitan RKN species; *M. incognia*, *M. javanica*, and *M. arenaria* (Hajihassani et al. 2019, Thies & Fery, 2000). Previous comparisons of *M. floridensis* isolates from Florida have shown that some isolates were able to overcome the *N* resistance gene in the pepper cultivar ‘Charlston Belle’ (Stanley et al., 2009). However, it is still unclear whether *M. floridensis* can overcome the other RKN resistance genes in pepper.

In this study, we set out to characterize the effectiveness of known RKN resistance genes in pepper against *M. floridness* by screening a panel of isogenic lines containing different resistance genes alongside a USDA line, PA 136, that was considered universally susceptible to all RKN species. Our results provide new insights into the virulence profile of *M. floridensis*.

## Materials and Methods

### Preparation of nematode inoculum:

The pure culture of *M. floridensis* obtained from Dr. Desaeger's lab at the University of Florida, Wimauma was maintained in a growth chamber on tomatoes (*Solanum lycopersicum* L., cv. Rutgers, Seedway, Hall, NY). Nematode eggs were extracted by agitating tomato roots in 0.6% NaOCl for 4 min, as described by Hussey and Barker (1973). The extracted eggs served as inoculum for the experiments.

### Pepper genotypes:

We screened eight pepper lines across three nematode bioassays (Table 1). Five of these pepper lines (‘Charleston Belle’, HDA 149, HDA 330, PM 217, and PM 687) were known to contain individual RKN resistance genes, and were included in all three bioassays alongside USDA pepper line PA 136 as a control. PA 136 was released as a universal RKN susceptible control line (Dukes & Fery, 1992). However, after observing resistance in PA 136 in the first bioassay, we switched our susceptible control to ‘Charleston Belle’ which had previously been shown to be susceptible to *M. floridensis* (Stanley et al., 2009). To further test the resistance in PA 136, in the second two bioassays we included two additional pepper lines; ‘Carolina Wonder’, another cultivar carrying the *N* resistance gene, and an F1 hybrid of ‘Carolina Wonder’ X PA 136.

### Nematode Bioassays:

Three bioassays were conducted in both space and time. The first experiment was carried out in a growth chamber at Clemson University in Clemson and included six pepper lines ‘Charleston Belle’, HDA 149, HDA 330, PM 217, PM 687, PA 136 (Table 1). The second experiment was performed in a greenhouse at Clemson University in Clemson and included all eight pepper lines ‘Charleston Belle’, HDA 149, HDA 330, PM 217, PM 687, PA 136, ‘Carolina Wonder’, ‘Carolina Wonder’ X PA136 F1 Hybrid (Table 1). The third experiment was conducted in a greenhouse at the USDA Vegetable Laboratory in Charleston, South Carolina and also included all eight pepper lines (Table 1). Six-week-old pepper seedlings were transplanted into 15-cm top diameter plastic pots. Each pot received 1.5 kg of sandy loam soil steam sterilized in four cycles of 123 °C for 45 min prior to use. Each experiment was established as a randomized block design with either four replications (first and second experiments) or ten replications (third experiment). Soils were infested 24 hr after transplanting by pipetting aqueous suspensions of 10,000 *M. floridensis* eggs into three depressions arranged into a triangular pattern, 0.5 cm diam. × 5 cm deep as described by Khanal et al. (2018). Plants were checked for water requirements daily and watered when required. Weekly fertilization (MiracleGro®, N-P-K 24-8-16), and standard chemical-based insect management practices were conducted.

**Table 1:** Pepper genotypes and their corresponding gene(s) for resistance employed in the current study.

**Pepper genotype**	**Known RKN resistance gene(s)**	**Reference**
‘Charleston Belle’	*N*	Fery et al., 1998
HDA 149	*Me3*	Djian-Caporalino et al., 2007
HDA 330	*Me1*	Djian-Caporalino et al, 2007
PM 217	*Me1, Me2*	Djian-Caporalino et al., 2007; Berthou et al., 2003
PM 687	*Me3, Me4*	Djian-Caporalino et al., 2007
PA 136	None	Dukes et al., 1992
‘Carolina Wonder’ (CW)	*N*	Fery et al., 1998
CW X PA136, F1 Hybrid	*N*	This study

Each experiment was terminated two months after inoculation. Individual plant roots were separated from the soil in pots and gently washed with tap water and blot dried with paper towels followed by weighing. The first two assays conducted at Clemson University scored galling using the root gall index (GI), as a measure of disease severity, were recorded using the root-knot rating chart of Bridge and Page (1980) in a scale of 0 to 10 (0 = no galls, 5= 50% roots infested, 10 = completely galled). The third assay conducted at the USDA scored galling using a percent root system galling (Ruter et al., 2021). For all three assays nematode eggs were extracted from individual root systems on the day of termination by agitating roots in 0.6% commercial bleach (NaOCl) for 4 min to dislodge eggs from egg masses (Hussey and Barker, 1973). Extracted eggs were enumerated within 24 hr of extraction using a stereoscopic microscope (Martin Microscope Company, Easley, SC) at 40× magnification. The average growth chamber temperatures and relative humidity during the study period were 28 ± 6 °C, and 45 ± 4%, respectively. The average temperatures and relative humidity during the first greenhouse study period were 27 ± 5 °C, and 45 ± 10%, respectively. The average temperatures during the second greenhouse study conducted at the USDA were 29 ± 5°C, and the relative humidity was 70 ± 10%.

### Data analysis:

Nematode reproduction, and root galling data from each experiment were analyzed separately in JMP PRO 16.2 (SAS, Cary, NC) because of the presence of significant experiment and treatment interactions. Residual analysis was conducted and outliers, if present, were excluded from subsequent analyses. Prior to mean comparisons using a one-way analysis of variance, data were subject to Goodness-of-Fit test for assessment of normality using Shapiro-Wilk and Anderson-Darling test. Non-normal data were subject to either *log* transformation or linked to Poisson distribution with *log* link based on the best fit statistics using Generalized Linear Mixed Model (GLMM). Treatments were used as fixed effects and replications were used as random effects. *Post-hoc* pairwise comparisons of treatment means were conducted using Student's *t*-test at *P* < 0.05. Treatment means presented in figures and table are back transformed values.

## Results

**Figure 1:**
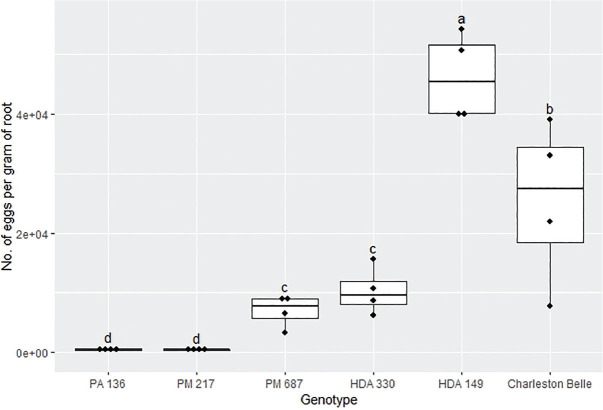
Reproduction of *Meloidogyne floridensis* on pepper genotypes in the growth chamber experiment conducted at Clemson University. Data represent the distribution of four replications. Tukey style box and whisker plots are presented with individual dot points representing scores from individual plants. Treatments sharing a common letter are not significantly different according to Student's *t*-test (*P* ≤ 0.05). The middle crossbar represents the median, the outer crossbars represent the 25^th^ and 75^th^ quartiles and the whiskers represent 3/2 times the interquartile range of the data.

**Figure 2:**
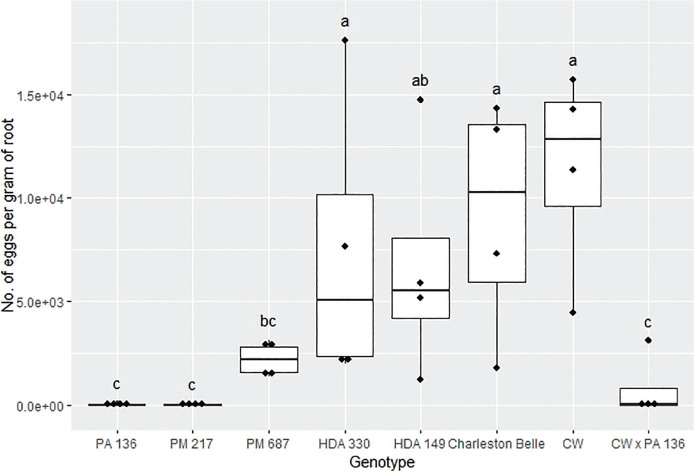
Reproduction of *Meloidogyne floridensis* on pepper genotypes in the first greenhouse experiment conducted at Clemson University. Data represent the distribution of four replications. Tukey style box and whisker plots are presented with individual dot points representing scores from individual plants. Treatments sharing a common letter are not significantly different according to Student's *t*-test (*P* ≤ 0.05). The middle crossbar represents the median, the outer crossbars represent the 25^th^ and 75^th^ quartiles and the whiskers represent 3/2 times the interquartile range of the data. CW refers to cultivar ‘Carolina Wonder’.

**Figure 3:**
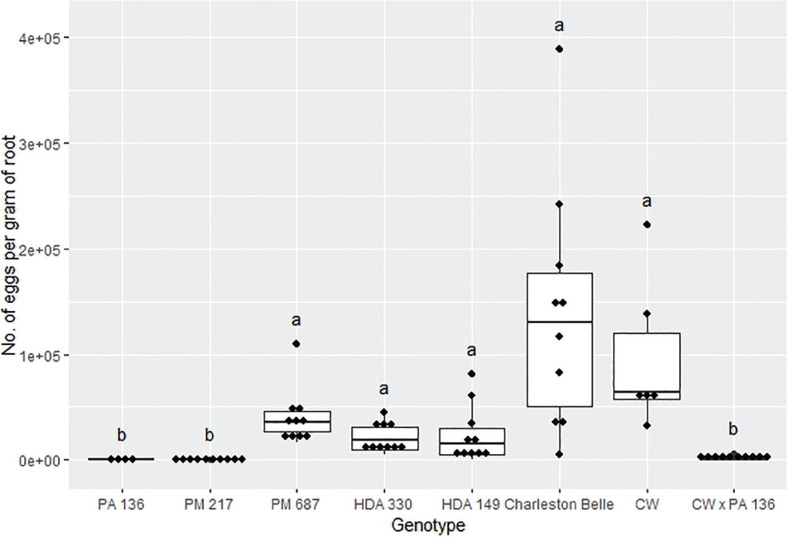
Reproduction of *Meloidogyne floridensis* on pepper genotypes in the first greenhouse experiment conducted at the USDA. Data represent the distribution of ten replications unless otherwise noted*. Tukey style box and whisker plots are presented with individual dot points representing scores from individual plants. Treatments sharing a common letter are not significantly different according to Student's *t*-test (*P* ≤ 0.05). The middle crossbar represents the median, the outer crossbars represent the 25^th^ and 75^th^ quartiles and the whiskers represent 3/2 times the interquartile range of the data. CW refers to cultivar ‘Carolina Wonder’. *Due to low seed germination two lines had less than ten replicates PA 136 had four replicates and CW had six replicates.

The reproduction of *M. floridensis* varied significantly among the pepper genotypes (*P* < 0.02) in all three bioassays despite the fact that all three were conducted in different environments and at different institutions (Figures 1, 2, and 3). However, the range of nematode reproduction was markedly different between the three experiments; ranging from 443 to 46,170 eggs/g root in the growth chamber experiment, 30 to 11,274 eggs/g root in the first greenhouse experiment, and 45 to 139,607 eggs/g root in the second greenhouse experiment. Despite the differences, in environments and laboratories we observed similar trends between the pepper genotypes across three experiments. In all three experiments, PA 136 and PM 217 supported the least nematode reproduction (Figures 1, 2, and 3), while ‘Charleston Belle’, HDA 149, HDA 330, and PM 687 in all three experiments supported significantly greater nematode reproduction compared to PA 136 and PM 217. Though it was only included in the two greenhouse experiments, ‘Carolina Wonder’ also supported significantly higher nematode reproduction than PA 136, PM 217, and its F1 hybrid (‘Carolina Wonder’ x PA 136). While the reproduction of *M. floridensis* on the F1 hybrid was statistically similar to that on PA 136 and PM 217.

**Figure 4:**
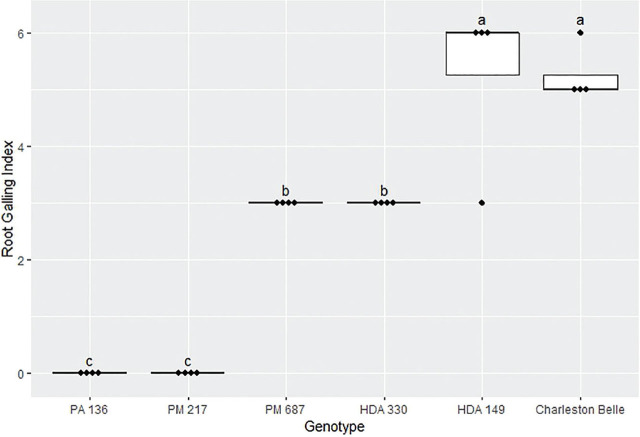
Root galling caused by *Meloidogyne floridensis* on pepper genotypes in the growth chamber experiment. Data represent the distribution of four replications. Tukey style box and whisker plots are presented with individual dot points representing scores from individual plants. Treatments sharing a common letter are not significantly different according to Student's *t*-test (*P* ≤ 0.05). The middle crossbar represents the median, the outer crossbars represent the 25^th^ and 75^th^ quartiles and the whiskers represent 3/2 times the interquartile range of the data. CW refers to cultivar ‘Carolina Wonder’.

**Figure 5:**
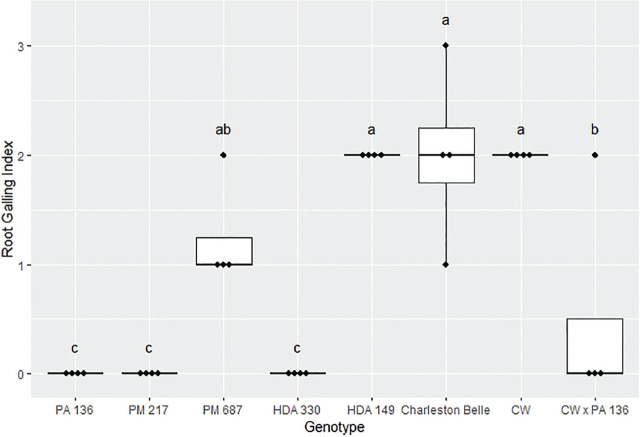
Root galling caused by *Meloidogyne floridensis* on pepper genotypes in the first greenhouse experiment conducted at Clemson University. Data represent the distribution of four replications. Tukey style box and whisker plots are presented with individual dot points representing scores from individual plants. Treatments sharing a common letter are not significantly different according to Student's *t*-test (*P* ≤ 0.05). The middle crossbar represents the median, the outer crossbars represent the 25^th^ and 75^th^ quartiles and the whiskers represent 3/2 times the interquartile range of the data. CW refers to cultivar ‘Carolina Wonder’.

**Figure 6:**
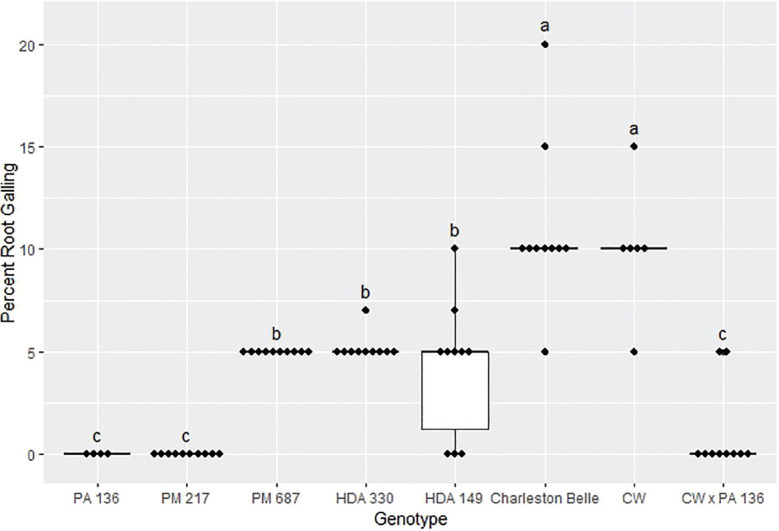
Root galling caused by *Meloidogyne floridensis* on pepper genotypes in the first greenhouse experiment conducted at the USDA. Data represent the distribution of ten replications unless otherwise noted*. Tukey style box and whisker plots are presented with individual dot points representing scores from individual plants. Treatments sharing a common letter are not significantly different according to Student's *t*-test (*P* ≤ 0.05). The middle crossbar represents the median, the outer crossbars represent the 25^th^ and 75^th^ quartiles and the whiskers represent 3/2 times the interquartile range of the data. CW refers to cultivar ‘Carolina Wonder’. *Due to low seed germination two lines had less than ten replicates PA 136 had four replicates and CW had six replicates.

The amount of galling on pepper roots was also significantly influenced by the genotype (*P* < 0.01) as presented in Figures 4, 5, and 6. The mean gall index in the growth chamber experiment ranged from 0 to 5.3, those in the first greenhouse experiment ranged from 0 to 3.5, while the mean percentage root system galled in the third experiment ranged from 0% to 11%. Line PA 136 and PM 217 showed the least damage in both experiments, receiving a mean galling score of 0 in all three bioassays. The rest of the genotypes had a significantly greater gall index, with HDA 330 in the in the first greenhouse experiment being the exception. While PM 687 and HDA 330 in the growth chamber experiment and the second greenhouse experiment had significantly greater galling scores relative to the PA 136 control, these lines had significantly lower galling scores relative to ‘Charleston Belle’ and HDA 149. ‘Charleston Belle’, HDA 149, PM 687 and ‘Carolina Wonder’ had statistically similar gall indices among themselves, however, their galling scores were significantly greater than the PA 136 control. In the first greenhouse assay, the F1 hybrid ‘Carolina Wonder’ x PA 136 had significantly greater galling than the PA 136 control but significantly lower than those of ‘Charleston Belle’, HDA 149, and ‘Carolina Wonder’ in both greenhouse assays, while PA 136 and PM 217 showed no galling in any of the three experiments.

## Discussion

Our results were consistent across three bioassays conducted in three different environments by two different labs. Although the average *M. floridensis* reproduction was markedly different between these three experiments, the relative differences between the pepper lines in each experiment were remarkably similar across the different environments. In all three experiments, lines PA 136 and PM 217 showed a high level of resistance to *M. floridensis,* displaying significantly less reproduction and galling compared to the other inbred pepper lines. Moreover, the known *M. floridensis* susceptible line ‘Charleston Belle’ showed a relatively high level of infection across all three experiments. Taken together these data provide strong evidence that the results of these three experiments are consistent and reliable.

Our results also confirm those of Stanley et al. 2009, who demonstrated that the RKN resistant ‘Charleston Belle’ (carrying the *N* resistance gene) was susceptible to *M. floridensis.* Our results further demonstrate that *M. floridensis* is virulent against additional RKN resistance genes in pepper, including *Me1* and *Me3*. These same resistance genes have been shown to be highly resistant to several other Clade I species including *M. incognita*, *M. javanica*, *M. arenaria*, as well as *M. haplanaria* (Hajihassani et al., 2019). This distinctive virulence profile is not altogether surprising given the fact that *M. floridensis* has already been reported to be virulent against several other RKN resistance genes, including the *RMia* gene in peach, the *RMja* gene in almond, the *Mi1.2* gene in tomato, and the *N* gene in pepper (Stanley et al., 2009; Maquilan et al. 2018; Marquez et al., 2023b). Our results further highlight the differences between *M. floridensis* and its close relatives and stress the need for additional research into the underlying molecular determinants of these parasite-host interactions. *Meloidogyne floridensis* has been shown to be very closely related to the other *M. incognita* group species (Szitenberg et. al., 2017), but in contrast to its asexual relatives it has retained diploidy and is currently the only known meiotically parthenogenic species within clade I. It is notable that other clade I RKN species such as *M. enterolobii* have also been shown to be virulent against these same resistance genes in pepper (Rutter et al., 2021, Brito et al., 2007). However, both because of its close relation to the *M. incognita* group species and comparatively simple genome, *M. floridensis* could serve as an important outgroup for identifying the molecular determinants of virulence against different resistance genes.

We were surprised to find that pepper lines PA 136 and PM 217 were highly resistant to this *M. floridensis*. The resistance in PA 136 was particularly surprising given that it was originally released as a universally RKN susceptible pepper line (Dukes and Fery, 1992), and has never been reported to be resistant to any other RKN species. To further confirm resistance in PA 136, we also tested an F1 hybrid ‘Caroliana Wonder’ X PA 136 in our two greenhouse experiments and found that this hybrid also displayed a high level of resistance compared to ‘Caroliana Wonder’. These results indicate that PA 136 contains an uncharacterized source of resistance that is specific against *M. floridensis* and has a dominant inheritance pattern. The second *M. floridensis* resistant line was PM 217, has been reported to carry a second resistance gene, *Me2,* which confers species-specific resistance against *M. hispanica* (Berthou et al., 2003). Though it is possible that *Me2* also confers resistance against *M. floridensis* it seems equally likely that it may carry a separate resistance gene. Additional investigation is needed to determine whether PA 136 or PM 217 carry the same or different sources of resistance against *M. floridensis*, and how widespread this resistance is across cultivated pepper lines where this nematode is present.

*Meloidogyne floridensis* has been shown to have a high level of inter-isolate variability. Different *M. floridensis* isolates were shown to have different pathotypes on RKN resistant pepper and tomato (Marquez et al., 2023b; Stanley et al., 2009). Similarly, there has been substantial inter-isolate variability reported for sexually reproducing RKN species such as *M. chitwoodi* (Berthou et al., 2003). Substantial variability among geographic isolates of other nematode genera have also been reported suggesting inter-isolate variability is widely present in sexually reproducing nematodes (McGawley et al. 2010, Khanal et al., 2018, 2019; Kularathna et al., 2019). Given the documented pathogenic variability within *M. floridensis,* further study is needed to determine how broadly effective these new sources are against different isolates.

To date, *M. floridensis* has been reported in four states, and in each case has been found infecting RKN resistant crop varieties. However, it is still not entirely clear how much of a threat this species poses to vegetable crops including pepper. Recent field surveys in GA and CA have found *M. floridensis* in regions known to have a history of peach cultivation (Marquez et al., 2023). Indeed, the peach and almond industries are reported to be under serious threat from this nematode (Brito et al. 2015, Westphal et al. 2019). Both because of its broad host range and the difficulty in distinguishing it from the *M. incognita* group species (Smith et al, 2015), it's possible that this nematode has been misidentified and may be more widespread than previously thought. Additionally, a predictable increase in virulence of *M. floridensis* at higher soil temperatures suggests crop damage from this nematode species will likely be greater in the future of global soil warming (Khanal and Land, 2023).

In summary, our results expand on previous reports of virulence, by showing that *M. floridensis* can overcome both the *Me1* and *Me3* genes in pepper. Interestingly, we also found an as yet uncharacterized source of species-specific resistance against this nematode in pepper lines PA 136 and PM 217. Follow up studies will be needed to determine how broadly *M. floridensis* resistance is conserved within commercially cultivated pepper varieties, and how effective this resistance is against variable isolates of this species. However, with this additional data on the virulence of *M. floridensis*, and the novel sources of resistance we have identified, we hypothesize that it will be possible to develop more broadly RKN resistant pepper varieties to help manage this nematode. Because the current nematode management practices rely heavily on the use of chemical nematicides which are detrimental to the environment and human health, cumbersome to apply and unable to provide season-long protection against nematodes (Khanal et al., 2018b, 2021, 2022; Khanal and Desaeger, 2020), the development of RKN resistant pepper varieties can help us drive towards sustainable agriculture.
